# “Khotla Bophelong Bo Botle”: a gathering of men for health

**DOI:** 10.1002/jia2.25511

**Published:** 2020-06-26

**Authors:** Stacie C Stender, Aleisha Rozario

**Affiliations:** ^1^ Jhpiego Cape Town South Africa; ^2^ Johns Hopkins University Bloomberg School of Public Health Baltimore MD USA; ^3^ Jhpiego Lesotho Maseru Lesotho

**Keywords:** primary healthcare, male clinic, chronic disease, differentiated care, HIV care continuum

Person‐centered care through integration of services and engaging clients in their own care is essential to achieving epidemic control of HIV. Verticalization of services along the care continuum contributes to inequities in access to care and leads to stigma and discrimination [1, 2]. Our experience implementing an integrated model of primary healthcare (PHC) services at Scott Hospital in Lesotho show that even in a country where one in four adults are living with HIV and substantial national and international resources have been expended over the past 15 years, clients want and expect holistic care.

Men in Lesotho are rarely first in line at health clinics and often do not seek healthcare until their condition becomes severe. PHC, the foundation of service delivery in the country, largely caters to women of reproductive age and children under five years of age; the majority (80%) of healthcare providers are women. While HIV prevalence in Lesotho is substantially higher among women than men 15‐49 years of age (28.8% vs. 18.5%), men living with HIV are less likely to know their status than women or be virally suppressed [3, 4, 5]. The 2016 Lesotho Population‐Based Impact Assessment revealed that men 35‐49 years of age had the highest incidence of HIV at 2.7%, higher than among girls 15‐24 years of age (1.6%) [5]. The inequities related to health information and care goes beyond HIV services. More than half of men 15‐49 years of age who participated in the Demographic Health Survey of 2014 had never had their blood pressure measured (58.5%), and men were less likely to seek treatment when experiencing symptoms associated with tuberculosis, the second leading cause of death in Lesotho [6].

In June 2016, Jhpiego collaborated with the Ministry of Health and Scott Hospital in Morija to transition an outpatient department (OPD) offering voluntary medical male circumcision into a “male clinic” offering comprehensive outpatient services for men, drawing on Jhpiego’s model of continuous engagement of men in defining their own healthcare needs. Prior to the establishment of the male clinic, men living with HIV seeking routine care queued with women and children in the general OPD of the hospital where two rooms are allocated for HIV services; men living with other chronic diseases or seeking acute care services queued in general OPD. In early 2016, the idea of a “male clinic” model was not readily accepted but its success rested on the foundation that it was designed with male clients to define, attract and comprehensively meet the needs of Basotho men, an underserved population in the health sector. The model of care is based upon a Theory of Change that takes into consideration cultural and gender norms and burden of disease in the country (Figure 1). Principles of the clinic are to address client needs holistically, providing comprehensive, integrated, person‐centered care, to address stigma and improve health outcomes. Based on continuous feedback by clients, the clinic is a specific space for men and is predominately staffed by male healthcare professionals, expanding services offered to focus on the person; scheduling appointments, and making every effort to have clients seen by the same provider at each visit. It is not a clinic for men living with HIV; it is a clinic where any man can access essential healthcare services – acute or chronic – in an environment that facilitates peer support.

**Figure 1:**
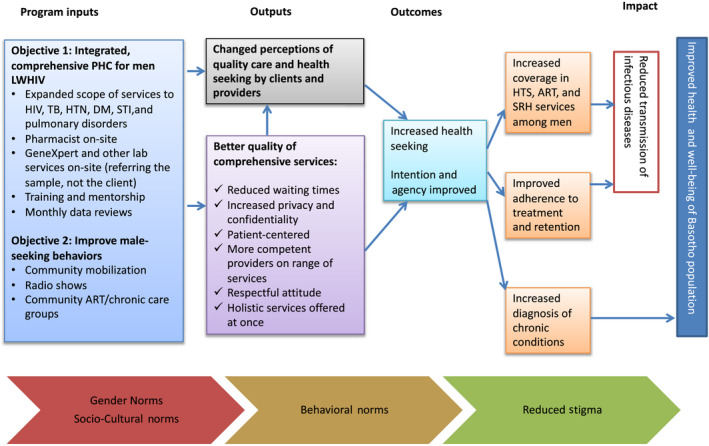
Theory of change for engaging and maintaining men in care.

Within the first six months of the clinic opening, evolving insights from men led to the refinement of the model of care, including the branding of the male‐centered services as “Khotla” meaning “where men gather,” a term that has deep cultural roots in Lesotho, eliciting a sense of confidentiality and importance among men. The longer phrase, “Khotla Bophelong Bo Botle,” specifically means “A gathering of men for health” in Sesotho. Today, key differences in care offered at Khotla compared to typical antiretroviral therapy (ART) and PHC clinics across the country include: reserved space and time for services offered to only men; higher male/female provider ratio; flexible routine clinic days (Monday to Saturday); and continuous consultation with clients to redefine services in order to evolve the model of care.

The average age of clients served is 48 years, and men come from all corners of this small, mountainous country. A mapping of clients enrolled in longitudinal care (men living with HIV and/or diabetes, hypertension or other chronic disease) in 2016/2017 revealed that men from all 10 districts travelled to Khotla at Scott Hospital despite decentralized ART and PHC services. Person‐centered respectful healthcare services need to be standard of care at the most decentralized level to ensure no one is left behind. In June 2017, the Ministry of Health began the expansion of the male clinic model to additional facilities across the country in collaboration with various partners, and as of September 2019 there were 20 “male clinics,” expanding essential care to men.

The Khotla model of care has shown to reach men who perhaps would not otherwise have been reached with essential healthcare services, as evidenced by the broad geographical distribution of clients served. The highest HIV incidence in Lesotho is among this “differentiated population” [7].

Implementation of comprehensive care necessitates expansion of PHC services for the population being served, and has been limited by the inability to address male‐specific needs including chronic, non‐communicable conditions that are not prioritized in health funding. To close the gender gap in HIV testing and treatment, differentiated care for integrated, quality, male‐centered clinical services must be scaled up with fidelity and healthcare needs of men – beyond HIV – have to be considered to keep clients engaged in lifelong healthcare. Context is fundamental to reaching men, as there are different social and cultural barriers and enablers that impact demand for and offering of services.

## Competing interests

Neither author has a conflict of interest.

## Authors’ contributions

SS contributed substantially to the concept and design of the model; AR, Jhpiego/Lesotho Country Director, has overseen project implementation of the model and co‐wrote the Viewpoint.

## Abbreviations

ART, antiretroviral therapy; OPD, outpatient department; PHC, primary healthcare.
